# Atypical Abdominal Ulcer Relapse of Granulomatosis With Polyangiitis Following Renal Transplantation With Bilateral Papillary Renal Cell Carcinoma: A Case Report

**DOI:** 10.7759/cureus.107283

**Published:** 2026-04-18

**Authors:** Mohammad Alkofahi, Ahmad Jalil, Ala Alkofahi, Punith Chirumamilla, Kirk Eddleman

**Affiliations:** 1 Internal Medicine, Baptist Memorial Hospital-North Mississippi, Oxford, USA; 2 Rheumatology, Baptist Memorial Hospital-North Mississippi, Oxford, USA

**Keywords:** anca associated vasculitis, cutaneous ulcer, end stage renal disease, granulomatosis with polyangiitis, post transplant relapse, renal cell carcinoma, renal transplantation, rituximab therapy

## Abstract

Granulomatosis with polyangiitis (GPA) is a necrotizing small- to medium-vessel vasculitis primarily affecting the respiratory tract and kidneys, with cutaneous involvement recognized but less commonly presenting as deep ulcerative lesions. GPA may progress to end-stage renal disease (ESRD), sometimes requiring renal transplantation, while long-term immunosuppression is associated with an increased risk of malignancy, including renal cell carcinoma (RCC). We present a 30-year-old man diagnosed with GPA at age 19 after presenting with hemoptysis and renal failure, with positive cytoplasmic antineutrophil cytoplasmic antibodies (c-ANCA) and cavitary pulmonary nodules. Despite induction and maintenance immunosuppressive therapy, he progressed to ESRD and underwent renal transplantation in 2019. In 2022, surveillance imaging revealed synchronous bilateral papillary RCC in his native kidneys, requiring bilateral nephrectomy. In 2023, he developed a painful periumbilical abdominal ulcer associated with systemic inflammation, and a biopsy confirmed ANCA-associated vasculitis. He was treated with rituximab, glucocorticoids, and avacopan, resulting in complete ulcer resolution within six months. This case highlights the complex longitudinal course of GPA, including atypical cutaneous relapse after renal transplantation and the development of malignancy, and underscores the need for clinical suspicion for atypical relapse and long-term malignancy surveillance in high-risk patients.

## Introduction

Granulomatosis with polyangiitis (GPA) is a necrotizing granulomatous vasculitis involving small- to medium-sized vessels that classically affects the upper airway, lungs, and kidneys, often presenting with cavitary pulmonary nodules and rapidly progressive glomerulonephritis [1-4]. It is a rare disease with an estimated annual incidence of approximately 10-20 cases per million and a prevalence of 100-300 cases per million [3]. Although modern immunosuppressive therapy has significantly improved survival, up to 30% of patients progress to end-stage renal disease (ESRD) within five years of diagnosis [5].

Cutaneous involvement occurs in approximately 30-50% of cases and most commonly manifests as palpable purpura or nodules [6,7]. However, deep abdominal ulcerative lesions are uncommon and may mimic infection, malignancy, or pyoderma gangrenosum, posing a diagnostic challenge [6,7]. Such ulcerative presentations are rarely reported in post-transplant patients, where immunosuppression complicates the differential diagnosis and increases the risk of infection and malignancy, representing a diagnostically challenging and underreported clinical scenario. In addition, chronic immunosuppression and prior cyclophosphamide exposure increase the risk of malignancy, including renal cell carcinoma (RCC), particularly in patients requiring renal transplantation [8-11].

We report a decade-long disease course of GPA complicated by ESRD, renal transplantation, bilateral papillary RCC, and an uncommon abdominal ulcer relapse successfully treated with rituximab and avacopan, an oral C5a receptor inhibitor that blocks complement-mediated neutrophil activation, thereby reducing inflammation in antineutrophil cytoplasmic antibody (ANCA)-associated vasculitis and offering a steroid-sparing therapeutic approach [12-15].

## Case presentation

**Figure 1:**
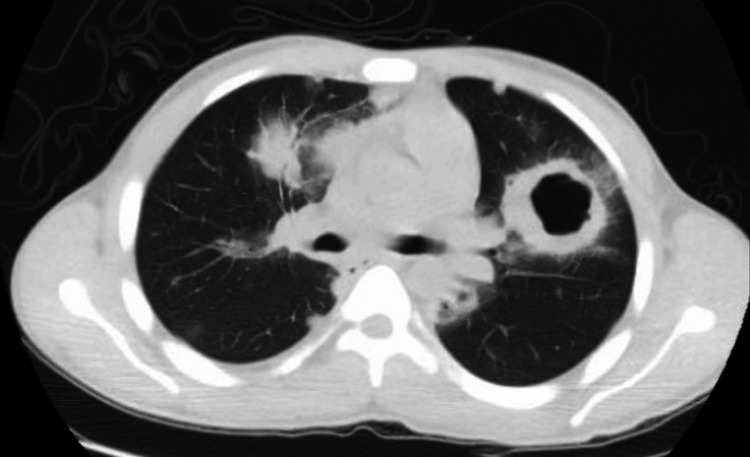
Axial CT chest demonstrating cavitary pulmonary nodules consistent with granulomatosis with polyangiitis at initial diagnosis.

A 19-year-old male initially presented in 2013 with acute hemoptysis and acute kidney injury. At the time of initial presentation, serum creatinine was 1.89 mg/dL, and urinalysis demonstrated 2+ proteinuria without evidence of active urinary sediment. CT imaging revealed multiple cavitary pulmonary nodules consistent with granulomatous vasculitis (Figure 1). Serologic testing was positive for cytoplasmic antineutrophil cytoplasmic antibodies (c-ANCA). A lung biopsy performed in 2013 demonstrated respiratory mucosa with ulceration and mixed acute and chronic inflammation, with ill-defined granulomatous inflammation and scattered multinucleated giant cells, consistent with GPA.

He received pulse-dose intravenous methylprednisolone (1 g daily for three days), followed by oral prednisone 60 mg daily and cyclophosphamide. Due to disease activity, he was later transitioned to azathioprine for maintenance and subsequently required rituximab for relapse. Renal function progressively worsened over time, with serum creatinine increasing from 2.3 mg/dL to over 10.5 mg/dL by 2018, ultimately resulting in ESRD requiring dialysis. After one year of dialysis, he underwent deceased-donor renal transplantation in 2019. His post-transplant course was uncomplicated, with no rejection episodes. Maintenance immunosuppression included tacrolimus, mycophenolate mofetil, and low-dose prednisone.

Pulmonary involvement demonstrated significant improvement over time. Initial imaging in 2013 revealed multiple bilateral cavitary nodules consistent with active vasculitis. Follow-up imaging in 2019 showed resolution of cavitary lesions with residual scarring, and subsequent imaging in 2023 demonstrated further stability with decreased lesion size and no new cavitary disease, consistent with clinical improvement.

**Figure 2:**
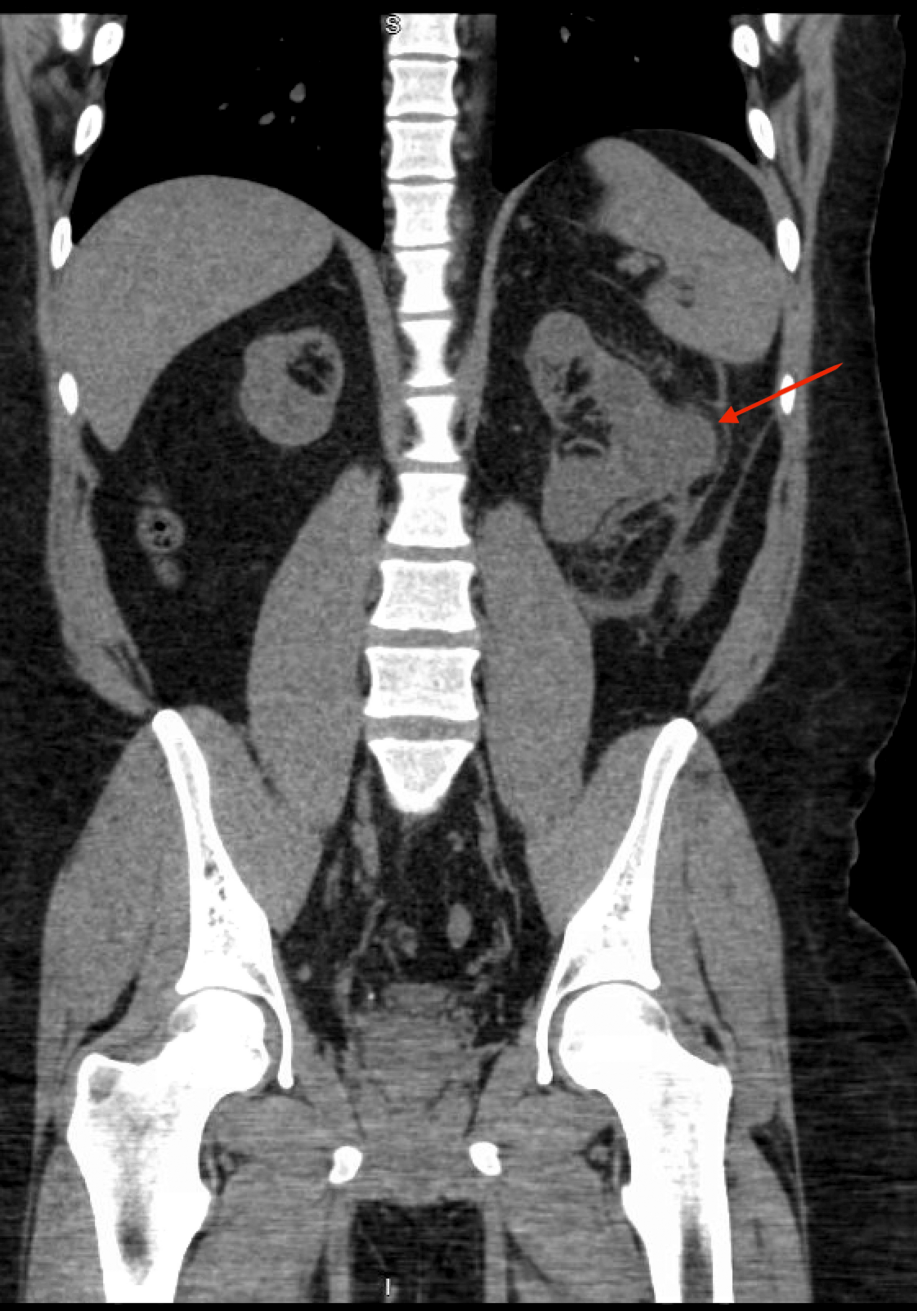
Coronal non-contrast CT abdomen and pelvis demonstrating a large mass in the left native kidney (arrow), concerning for malignancy. Subsequent MRI revealed additional bilateral renal lesions, which were later confirmed as papillary renal cell carcinoma on histopathology.

In 2022, surveillance CT imaging of the abdomen and pelvis identified a large indeterminate mass in the left native kidney measuring up to 6.3 × 6.6 × 7.8 cm, concerning for malignancy (Figure 2). Subsequent MRI of the abdomen, performed externally for further characterization, demonstrated multiple bilateral renal masses that were not consistent with simple cysts and were highly suspicious for RCC. Pathology following bilateral nephrectomy confirmed multifocal papillary RCC, nuclear grade 2, with the right kidney measuring 4.5 cm and the left 7.5 cm, staged as T2, with no evidence of metastasis.

**Figure 3:**
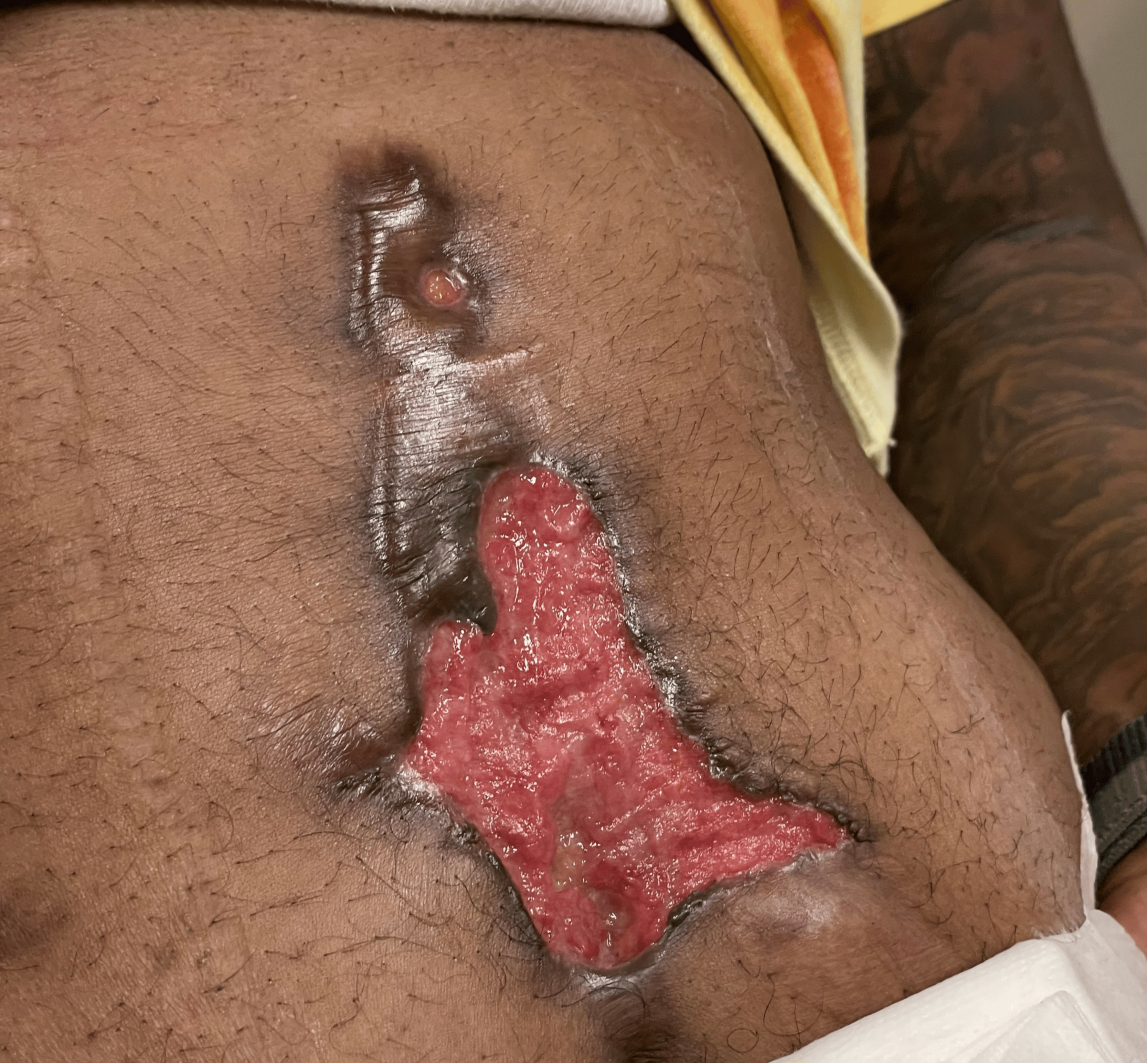
Abdominal ulcer at relapse demonstrating irregular erythematous granulating base and surrounding induration. Written informed consent was obtained from the patient for publication of this case report and accompanying images.

In 2023, he developed a painful ulcerative lesion over the periumbilical and lower midline abdominal wall associated with fever. Laboratory studies revealed an elevated erythrocyte sedimentation rate (ESR) of 57 mm/h and C-reactive protein (CRP) of 2 mg/dL (normal <0.3 mg/dL). Serologic testing demonstrated elevated c-ANCA titers (1:80) with anti-proteinase 3 (PR3) levels >8.0, consistent with active disease. A dermatologic biopsy demonstrated neutrophilic and granulomatous inflammation consistent with GPA; histopathologic images were not available for inclusion as the biopsy was performed at an external laboratory. No evidence of infection or malignancy was identified. On examination, the lesion measured approximately 8 × 5 cm with an irregular erythematous granulating base and surrounding induration (Figure 3).

**Figure 4:**
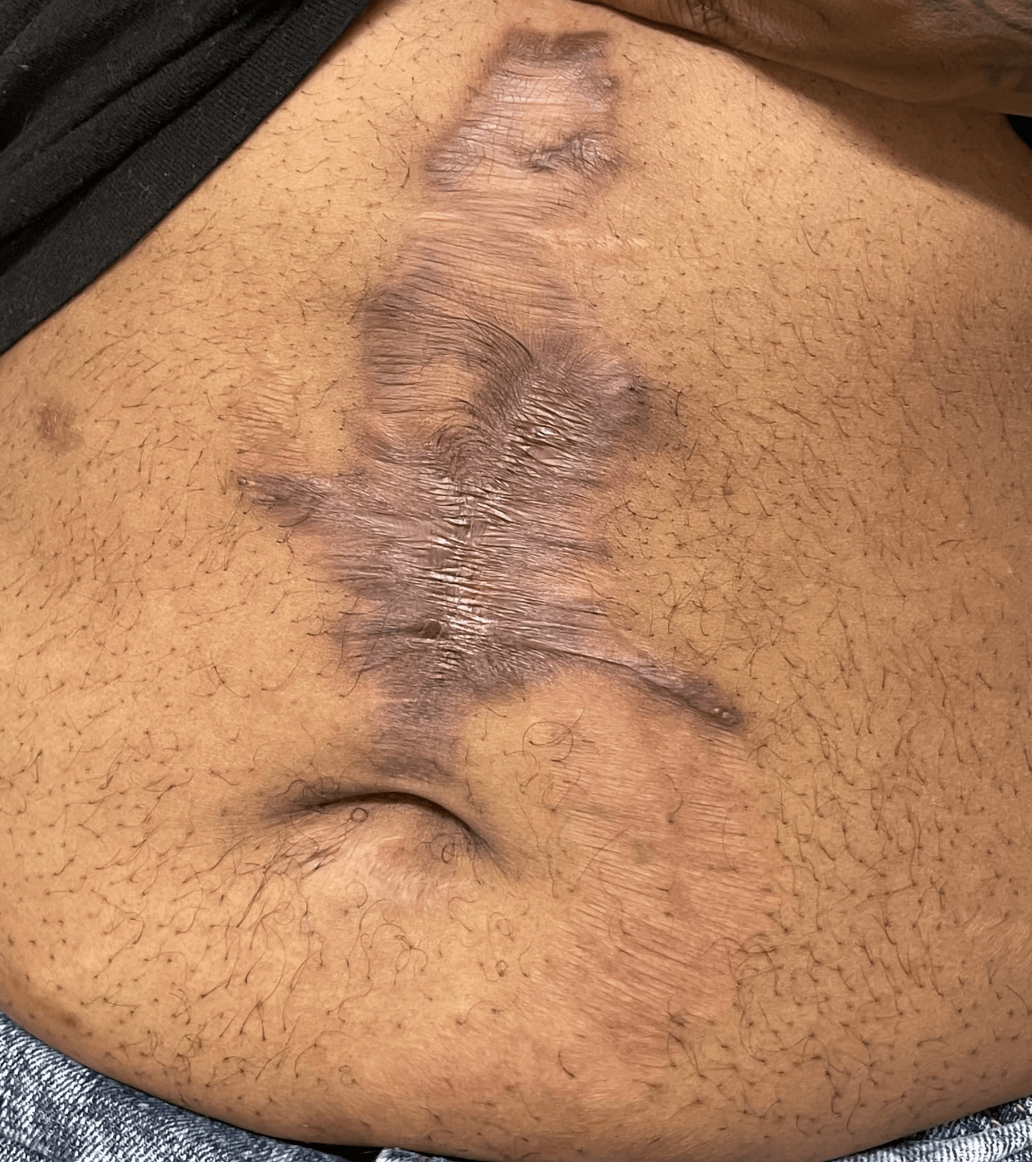
Complete epithelialization of the abdominal ulcer following rituximab and avacopan therapy with residual atrophic hyperpigmented scarring. Written informed consent was obtained from the patient for publication of this case report and accompanying images.

He was treated with rituximab (375 mg/m^2^ weekly for four doses), avacopan 30 mg twice daily, and prednisone initiated at 60 mg daily with a gradual taper. Over six months, the ulcer demonstrated progressive epithelialization with complete resolution, leaving a hyperpigmented atrophic scar (Figure 4).

As of 2025, he remains clinically stable on maintenance immunosuppression consisting of tacrolimus (Envarsus XR 1 mg daily), mycophenolic acid (Myfortic 180 mg three times daily), and prednisone 5 mg daily, used concurrently, with stable graft function, no episodes of rejection, and no additional systemic relapse.

## Discussion

GPA is a systemic necrotizing vasculitis that commonly involves the respiratory tract and kidneys, with cutaneous manifestations occurring in a subset of patients. While pulmonary and renal involvement are well recognized [1-3], atypical cutaneous relapse presenting as a large abdominal ulcer is rare. Cutaneous GPA occurs in approximately 30-50% of patients [6,7]; however, deep abdominal ulcerative lesions are uncommon and may mimic infection, malignancy, or neutrophilic dermatoses such as pyoderma gangrenosum, creating significant diagnostic uncertainty [6]. Such presentations are infrequently described, particularly in post-transplant patients, further emphasizing the atypical nature of this case. In this context, early tissue biopsy was essential in distinguishing vasculitic involvement from alternative etiologies and guiding appropriate immunosuppressive therapy.

Progression to ESRD remains a serious complication of GPA despite advances in immunosuppressive therapy [5,8]. Renal transplantation during disease remission is generally considered safe, with low relapse rates of approximately 0.02 episodes per patient-year [9]. However, cumulative exposure to cyclophosphamide and long-term immunosuppression contribute to an increased risk of malignancy [10,11]. Cyclophosphamide exposure has been associated with an increased risk of malignancy, particularly bladder cancer and other solid tumors [10,11]. RCC has been reported in patients with ANCA-associated vasculitis, particularly in the setting of prior cyclophosphamide exposure and chronic immunosuppression [10-12]. The development of synchronous bilateral papillary RCC in this patient highlights the need for ongoing malignancy surveillance in high-risk populations.

The use of rituximab in this relapse is supported by randomized controlled trials demonstrating its efficacy in both induction and maintenance of remission in ANCA-associated vasculitis [13,14]. In addition, avacopan, a selective C5a receptor inhibitor, has emerged as an effective steroid-sparing agent, as demonstrated in the ADVOCATE trial, achieving sustained remission while reducing glucocorticoid toxicity [15]. In this case, the addition of avacopan to rituximab was guided by the need for effective disease control while minimizing glucocorticoid exposure in a patient with relapsing, multisystem disease, particularly in the post-transplant setting. Avacopan targets complement-mediated neutrophil activation, a key pathway in the pathogenesis of ANCA-associated vasculitis. Emerging evidence supports the use of avacopan in combination with standard immunosuppressive therapies to optimize disease control and reduce treatment-related adverse effects [16]. No significant pharmacologic interactions have been reported between avacopan and calcineurin inhibitors such as tacrolimus; however, close clinical monitoring is recommended when combining these agents with other immunosuppressive therapies.

Pyoderma gangrenosum was considered in the differential diagnosis; however, the presence of granulomatous inflammation on biopsy and concurrent serologic evidence of active GPA supported vasculitic involvement. The patient’s established history of GPA, elevated c-ANCA titers (1:80) and anti-PR3 levels (>8.0), and exclusion of infectious and malignant etiologies further strengthened the diagnosis of GPA relapse over alternative causes.

A key limitation of this case is the absence of histopathologic images, as the biopsy was performed at an external laboratory despite attempts to obtain the original slides. However, the histopathologic report described neutrophilic and granulomatous inflammation, which, in the appropriate clinical and serologic context, supports a diagnosis of GPA. Histologically, GPA is characterized by necrotizing granulomatous inflammation with mixed inflammatory infiltrates and vasculitis. In contrast, pyoderma gangrenosum typically demonstrates a predominantly neutrophilic dermatosis without granulomatous inflammation or true vasculitis, while malignant ulcers show atypical malignant cells with invasive growth patterns. In this case, the integration of clinical history, serologic findings, and histopathologic features strongly favored vasculitic relapse over alternative diagnoses, particularly in the context of a post-transplant patient with relapsing multisystem disease.

This case underscores the importance of multidisciplinary long-term follow-up, as patients with GPA remain at risk for relapse, organ failure, and malignancy even many years after initial diagnosis. It also highlights the need for a high index of suspicion for atypical disease manifestations and the importance of integrating clinical, serologic, and histopathologic data to guide diagnosis and management in complex cases.

## Conclusions

This case highlights the complex, multisystem course of GPA and the potential for atypical cutaneous relapse even years after initial diagnosis. In this patient, immunosuppressive therapy was individualized based on relapsing disease in the post-transplant setting, with the use of rituximab and avacopan to achieve effective disease control while minimizing glucocorticoid exposure. This approach resulted in complete resolution of the ulcerative lesion.

This case underscores the importance of maintaining a broad differential diagnosis for ulcerative lesions in immunosuppressed patients, particularly in those with underlying vasculitis, where atypical presentations may mimic infection or malignancy. Early tissue biopsy and integration of clinical, serologic, and histopathologic data remain essential for accurate diagnosis and appropriate management. A limitation of this report is the absence of histopathologic images, as the biopsy was performed at an external laboratory, which may limit independent interpretation of the findings.
